# Reconceptualizing Peri‐Implantitis: Dual‐Factor Inflammation and Its Role in Advancing Biomaterial Coatings

**DOI:** 10.1111/cid.70108

**Published:** 2026-01-09

**Authors:** Daniela Moreira Cunha, Amanda Paino Santana, Mariana Martins Guerreiro, Akhilanand Chaurasia, Anton Sculean, Rafael Scaf de Molon, Erica Dorigatti de Avila

**Affiliations:** ^1^ Department of Dental Materials and Prosthodontics, School of Dentistry São Paulo State University (UNESP) Araçatuba Brazil; ^2^ Department of Diagnostic and Surgery, School of Dentistry São Paulo State University (UNESP) Araçatuba Brazil; ^3^ Department of Oral Medicine and Radiology King George's Medical University Lucknow India; ^4^ Department of Periodontology University of Bern Bern Switzerland

## 1 Microbial Dysbiosis as a Primary Trigger in Peri‐Implantitis: Etiology Versus Modifying Factors for Peri‐Implantitis

There is no doubt that peri‐implantitis is an inflammatory condition affecting the tissues surrounding dental implants, driven by a complex interplay between microbial dysbiosis and the host immune response [1]. Subclinical interactions between the dysbiotic biofilm and the host immune system are initiated largely by components of anaerobic Gram‐negative bacteria, most notably lipopolysaccharide (LPS), which predominates at diseased peri‐implant sites. LPS engages toll‐like receptor 4 (TLR4) on epithelial and immune cells, activating transcriptional pathways that upregulate proinflammatory mediators and establish a permissive environment for leukocyte recruitment [2]. Neutrophils, macrophages, and dendritic cells are rapidly mobilized and amplify tissue destruction through the release of cytokines and extracellular matrix‐degrading enzymes. Among these innate immune cells, macrophages play a particularly central role in peri‐implant disease pathogenesis, exhibiting a pronounced shift toward an M1‐dominant phenotype [3, 4]. Interestingly, macrophage polarization has also been implicated as a key pathogenic mechanism across a broad spectrum of chronic inflammatory diseases, including cancer, diabetes, atherosclerosis, and periodontitis [5, 6] supporting the notion that peri‐implantitis may share pathogenic pathways with, and potentially associate with, other local and systemic inflammatory conditions. M1‐polarized macrophages produce RANKL, reactive oxygen species, and matrix metalloproteinases, thereby promoting osteoclastogenesis and accelerating bone resorption. Neutrophils further contribute through membrane‐bound RANKL expression and heightened sensitivity to microbial cues. As disease progresses, adaptive immunity becomes similarly dysregulated, with helper T‐cells (CD4^+^ T)‐cell subsets, including Th1, Th2, Th17 cells, and regulatory T cells (Tregs), sustaining chronic inflammation and driving interleukin (IL‐)17‐mediated neutrophil recruitment and osteoclast activation. Tregs, characterized by expression of transcription factor forkhead box p3 (FOXP3), cluster of differentiation (CD)25, and the IL‐2 receptor, are markedly elevated in peri‐implantitis lesions and are typically associated with the production of IL‐10, tgf beta growth factor (TGF)‐β1, and IL‐35. While IL‐10 and TGF‐β1 function as baseline homeostatic anti‐inflammatory mediators, IL‐35 is induced under intense inflammatory pressure. The observed imbalance with elevated FOXP3 and IL‐35 coupled with reduced TGF‐β1 suggests a dysfunctional or unstable Treg phenotype unable to effectively restrain inflammation, thereby contributing to the progressive soft‐ and hard‐tissue destruction characteristic of peri‐implantitis [7].

In simple terms, a dysbiotic biofilm, an imbalanced and pathogenic microbial community adhered to the implant surface, acts as the primary trigger, initiating an inflammatory cascade. However, the nature, intensity, and chronicity of this inflammation are determined largely by host‐specific immune mechanisms. The immune system's role is pivotal: it not only mediates the initial response but also dictates whether inflammation remains localized as peri‐implant mucositis or progresses to peri‐implantitis, potentially culminating in significant bone loss and implant failure [8]. This raises an important conceptual question: if disease initiation requires both the presence of a dysbiotic biofilm and an individual's susceptibility, why is peri‐implantitis still described as a multifactorial disease rather than a dual‐factor condition, where both microbial and host factors act as necessary and interconnected etiologic drivers?

To address this, it is crucial to distinguish the core etiology of peri‐implantitis from the numerous modifying factors that influence its clinical expression and progression. While systemic conditions, lifestyle habits, and implant‐related design features can modulate the severity and trajectory of the inflammatory response, they do not constitute the primary triggers of the condition. The etiology of peri‐implantitis is defined by the convergence of two fundamental elements: (i) the presence of a dysbiotic biofilm, serving as the primary microbial driver; and (ii) host susceptibility, which is largely governed by individual genetic and immunological variability [9]. These host‐specific variations affect immune recognition mechanisms, the release of inflammatory mediators, and the efficiency of tissue repair, thereby contributing to the considerable interindividual variability observed in both disease susceptibility and clinical severity.

This conceptual tension becomes clearer when examining key direct factors consistently identified in the literature. Two stand out in particular: poor oral hygiene and history of periodontitis. Poor oral hygiene is the most controllable risk factor; inadequate plaque control promotes biofilm accumulation on and around implants, and biofilm is recognized as the primary driver of peri‐implant tissue inflammation [10]. Meanwhile, a history of periodontitis significantly increases susceptibility to peri‐implantitis due to heightened baseline inflammatory reactivity and a preconditioned immune environment that favors disease progression [8]. Together, these factors illustrate how microbial and host elements are not merely contributing variables but fundamental components whose interaction underpins the pathogenesis of peri‐implantitis.

## 2 Current Concepts of Peri‐Implantitis as a Multifactorial Condition

The current definition of peri‐implantitis classifies it as a multifactorial disease because, beyond susceptibility and biofilm presence, environmental/systemic factors and implant‐related characteristics can modulate the course of the disease. These variables often interact with each other and with host factors, creating an environment that facilitates both the onset and progression of peri‐implant pathology. Importantly, these influences are not always direct causes; instead, they modify disease risk, accelerate tissue destruction, or reduce the host's ability to contain infection.

In this context, several key factors have been identified as contributing to peri‐implantitis and can be categorized as local, systemic, and implant‐related, as previously described. Although these factors are not primary etiologic agents, they can significantly influence the onset, progression, and overall dynamics of the disease.

- Implant surface roughness—while the implant surface itself is not pathogenic, rougher textures promote more rapid and extensive biofilm accumulation compared with smoother surfaces. This mechanical feature can indirectly fuel inflammation and contribute to disease progression [11].
- Smoking—tobacco use compromises vascularization, reduces oxygen delivery, and impairs immune cell function. In the peri‐implant context, smoking also shifts the oral microbiome toward a more pathogenic composition, increasing both infection risk and healing delays [12].
- Diabetes—although a definitive causal relationship remains under investigation, evidence indicates that chronic hyperglycemia alters immune function, compromises wound healing, and may exacerbate peri‐implant inflammation [13].
- Keratinized mucosa (KM) width—A KM width < 2 mm has been associated with greater plaque accumulation, inflammation, and peri‐implant disease occurrence [14, 15]. Adequate KM acts as physical and immunological barrier, that is, as an indirect strategy to prevent peri‐implantitis [16].
- Implant–abutment connection design—different mechanical connections vary in their resistance to bacterial leakage, influencing the microbial environment at the implant interface [17].
- Excess cement—the presence of residual cement has been associated with peri‐implant disorders [18], as it can elicit a foreign body reaction and potentiate the inflammatory response [19], with bacterial colonization of the retained cement acting as the initiating factor [20].
- Genetic predispositions—polymorphisms in genes regulating the inflammatory response, such as: IL‐1 [21], TNF‐α, OPG, as well as the CD14‐159 C/T and TNF‐α‐308 A/G variants [22] have been associated with increased susceptibility to peri‐implantitis. These genetic variations may influence individual host responses and represent potential biomarkers for identifying patients at elevated risk [22].
- Occlusal overload—occlusal overload is associated with biomechanical complications, including fractures or loosening of the implant and its prosthetic components, which can compromise implant integrity and promote additional biofilm accumulation. It may also impair the implant–bone interface, enhance inflammatory responses leading to peri‐implant bone loss, and ultimately increase the risk of implant failure, particularly in the presence of poor oral hygiene [23, 24].
- Titanium particle release—although inconclusive, titanium particles have been implied in the peri‐implantitis progression [1]. Titanium particles may be released from implant surfaces following procedures that induce surface damage [25] and their release can be further promoted by pathogenic biofilms adhering to dental implant components. These biofilms can accelerate titanium ion exchange with saliva, thereby reducing the corrosion resistance of titanium and increasing surface roughness [26] factors that may help explain the higher concentrations of titanium particles reported at peri‐implantitis sites [27, 28, 29, 30]. The presence of titanium particles has also been associated with the activation of resident immune cells, leading to a localized inflammatory response that may sustain or exacerbate chronic inflammation [31]. This mechanism suggests that titanium particle dissemination could indirectly contribute to the progression of established peri‐implantitis.
- Iatrogenic causes—iatrogenic factors, such as: inadequate restoration–abutment seating, overcontoured restorations, and implant malpositioning, have been identified as potential risk indicators for peri‐implantitis, as these conditions can facilitate biofilm accumulation by hindering effective self‐performed oral hygiene [20].

## 3 Integrating Etiological Insights Into the Development of Bioresponsive Coatings for Peri‐Implantitis

A deeper understanding of peri‐implantitis, as a condition driven by the interplay between microbial dysbiosis and host‐specific immune susceptibility, has critical implications for designing next‐generation implant surface coatings. The dual‐factor concept of peri‐implantitis, which recognizes both bacterial biofilm formation and host susceptibility as essential etiologic components, has become an important framework for guiding clinical decision‐making and research. Clinically, it emphasizes the need for careful prosthetic and surgical planning to minimize iatrogenic contributors to biological complications. From a research perspective, it directs the development of innovative biomaterials and implant surface technologies aimed at combating infectious processes and modulating host responses. This framework has catalyzed development of implant surfaces with enhanced anti‐infective properties aimed at preventing initial biofilm formation, as well as systems capable of local antimicrobial delivery to support conventional debridement techniques in managing active disease [16]. These strategies reflect a broader therapeutic shift from traditional, passive implant surfaces toward bioresponsive materials that not only resist microbial colonization but also interact dynamically with the surrounding biological environment. Such advances have the potential to significantly reduce infectious burden while facilitating a more favorable host response, ultimately improving clinical outcomes in both prevention and treatment of peri‐implantitis.

Recently, we classified different coating strategies for the management of peri‐implantitis and clarified the indications for this type of system in treating the associated inflammatory condition [16]. For peri‐implantitis prevention, the patient is presumed to be in a clinically healthy state; however, considering the individual's inherent susceptibility and the role of biofilm as the primary trigger of the inflammatory response, this type of coating would function by repelling bacterial adhesion or exerting contact‐killing activity. From a biomaterial perspective, the reagents incorporated in the coating must withstand the hostile oral environment to maintain structural integrity and ensure long‐term functional viability.

From a treatment standpoint, current peri‐implantitis treatment focused predominantly on implant surface decontamination through mechanical and chemical biofilm removal and their byproducts to reduce the infectious process and, consequently, control the associated inflammatory response. Adjunctive systemic or local antimicrobials are frequently incorporated to enhance infection control and improve clinical outcomes. However, growing interest has emerged in the development of bioresponsive coatings that not only provide immediate control of the microbial burden but also modulate the host response, with the potential to mitigate or even reverse peri‐implant soft‐ and hard‐tissue destruction [32]. These coatings are engineered to incorporate drugs and control their release over time using stimuliresponsive material to react dynamically to external (temperature and light) or internal (pH variation, enzymatic activity, and redox potential) cues within the biological environment. Overall, studies in the bioresponsive coatings field have often overlooked critical questions concerning the specific area targeted and whether a given coating is designed for disease prevention or active treatment. Such conceptual ambiguities hinder the rational development and validation of coating technologies and ultimately limit their translation into predictable clinical applications.

As a responsive coating, it is expected to lose integrity over time, depending on the targeted stimulus and the nature of the materials used in its construction. In other words, the coating tends to degrade to release the incorporated agent, which situates its action within the context of an already established clinical disease. With regard to the coating construction, chemical cross‐linking processes and layer‐by‐layer (LbL) systems [33, 34, 35, 36] might act as responsive systems for releasing the loaded target agents.

Within the context of peri‐implantitis, bioresponsive coatings may serve as adjuvant therapeutic platforms by delivering antimicrobial or host‐modulating agents offering immediate and local action in a controlled manner against implant‐associated infections. Antimicrobial agents are designed to target a broad spectrum of microorganisms involved in implant‐related infections, such as antibiotics [34, 37] and metal nanoparticles [38]. In the case of immunomodulatory agents, this class of drugs acts to modulate the immune response, offering a more targeted approach by regulating the inflammatory response. Essentially, these drugs can help reduce the excessive inflammation that contributes to bone destruction, promote healing and tissue regeneration around the implant, and enhance the host's capacity to control bacterial challenges. Among the most studied immunomodulatory agents, statins and specialized proresolving mediators (SPMs) have shown robust anti‐inflammatory activity. Statins exert their effects by protecting the vascular endothelium from damage and dysfunction, displaying antioxidant activity through the scavenging of free radicals, and modulating immune pathways that regulate inflammatory responses [39]. SPMs, in turn, exhibit potent anti‐inflammatory and proresolution actions by promoting neutrophil apoptosis, recruiting resolution‐promoting monocytes, and enhancing bacterial clearance, largely by improving the capacity of phagocytes to engulf and eradicate pathogens at mucosal surfaces [40].

These emerging strategies raise a critical question: could targeting the inflammatory process itself represent a viable therapeutic pathway for peri‐implantitis? Pathogenic microorganisms and their metabolic byproducts initiate a destructive immune cascade, making inflammation control a central component of peri‐implantitis management. However, inflammation control alone is insufficient as a standalone intervention. Effective treatment requires a comprehensive, multimodal approach that integrates antimicrobial therapies to reduce bacterial load, mechanical disruption of biofilm, and, in advanced cases, surgical procedures to restore tissue architecture. Within this framework, immunomodulating agents, though promising for regulating host inflammatory responses, should not be employed as primary drug delivery systems for peri‐implantitis. Their systemic action lacks the localized, targeted efficacy required to directly address microbial burden and tissue destruction. Moreover, during active disease, the immediate priority is to control infection and physically disrupt biofilm at the implant site. Immunomodulators do not provide rapid antimicrobial effects or mechanical removal and are therefore insufficient as standalone interventions. Instead, they may serve as adjuncts to support immune regulation following infection control but are not appropriate for localized delivery systems intended to manage acute inflammation.

## 4 Conclusion and Future Research Directions

This commentary highlights the need for patient‐specific biomaterial innovations that address both microbial and host components of peri‐implant disease. Given the inherent variability in patient susceptibility, it is essential to understand individual immunological limitations when planning rehabilitative procedures within a clinical context. Currently, no commercially available coating exists that either prevents bacterial adhesion or functions as an adjuvant therapy for peri‐implantitis. As previously discussed by our research group [41], the discrepancy between the large volume of published studies on antimicrobial coatings field and the absence of clinical products clearly illustrates that preclinical investigations continue to face considerable barriers before translation to clinical setting. Although preclinical research is indispensable for providing detailed insights into chemical and physical modifications of biomaterials and the potential adverse effects of coatings, the persistent stagnation in clinical implementation likely reflects the inherent complexity of factors involved in antimicrobial coating development [41]. In revisiting the fundamental chemical principles underlying material construction and aligning them with real clinical needs, we identified the necessity of making the conceptual basis of surface modification more didactic. From this analysis, we established two foundational elements that must be clearly defined before initiating the development of any biomaterial: (1) a precise understanding of the disease and its etiology, and (2) a clearly articulated coating purpose. This framework emphasizes the importance of aligning disease stage with material design, particularly in high‐risk individuals. Effective prevention and treatment demand medical coatings engineered for distinct clinical objectives, each with specific physicochemical and biological properties. For prevention, coatings must: (i) resist mechanical fatigue and chemical degradation in the oral environment; (ii) maintain bioactive functionality over time. For therapeutic use in active disease, coatings must: (i) respond to environmental cues; and (ii) deliver localized antimicrobials to combat infection and support conventional debridement.

## Funding

This study was funded by the State of Sao Paulo Research Foundation (FAPESP, Brazil) (grant numbers: 2025/07203‐1 to D.M.C.; 2021/09434‐0 and 2018/20719‐3 to E.D.A; 2023/15750‐7 to R.S.M; 2025/02912‐4 to A.P.S and 2024/14871‐8 to M.M.G.).

## Conflicts of Interest

The authors declare no conflicts of interest.

## Data Availability

Data sharing not applicable—no new data generated or analyzed during the current study.
